# Chordomas and chondrosarcomas of the skull base: treatment and outcome analysis in a consecutive case series of 24 patients

**DOI:** 10.1186/s12957-021-02178-6

**Published:** 2021-03-09

**Authors:** Christopher Metcalfe, Jameel Muzaffar, Kevin Kulendra, Paul Sanghera, Simon Shaw, Amjad Shad, Natarajan Saravanappa, Alessandro Paluzzi, Shahzada Ahmed

**Affiliations:** 1 Regional Skull-Base Unit, Queen Elizabeth Hospital, University Hospitals Birmingham, Mindelsohn Way, Birmingham, B15 2GW UK; 2 Royal Stoke University Hospital, Newcastle Road, Stoke-on-Trent, ST4 6QG UK; 3 University Hospital Coventry & Warwickshire, Clifford Bridge Road, Coventry, CV2 2DX UK

**Keywords:** Chondrosarcoma, Chordomas, Skull base, Endonasal, Transnasal, Proton beam therapy

## Abstract

**Background:**

We present our 9-year consecutive case series of skull base chordomas and chondrosarcomas from a UK tertiary referral centre, discussing treatments offered and outcomes. This was carried out to improve understanding around current treatment and to better inform the management of future patients.

**Methods:**

Consecutive case series over a 9-year period (2007–2016). Retrospective data analysis from the electronic skull base multidisciplinary team database and the digital patient records at a UK tertiary referral centre

**Results:**

Twenty-four patients were identified (11 chordomas, 13 chondrosarcomas, mean age 52). Nineteen had proton beam therapy (PBT) postoperatively; two had intensity-modulated radiotherapy; two had no further treatment. One patient was lost to follow-up. All chordomas were resected via a transnasal endoscopic approach. Of the 19 patients undergoing resection with PBT, 13 were disease free at latest follow-up, and six patients had local recurrence, of which two died (mean follow up 7.4 years). Of the three patients treated with surgery then IMRT/TomoTherapy, one died 4 years post-treatment, and the other two are alive after 4 and 5 years of follow-up respectively. Of the two patients treated with surgery alone, one was lost to follow-up, and the other is alive after more than 8 years. Chondrosarcoma 5-year survival was 91.6%, and chordoma 4-year survival was 75%.

**Conclusion:**

Skull base chordomas and chondrosarcomas can be challenging to resect, and most cases require adjuvant therapy to achieve control. Where complete resection is not possible, it is critical to undertake sufficient resection to permit high-dose radiation.

## Background

Chordomas and chondrosarcomas are rare bony tumours of the skull base with a combined annual incidence of approximately 1/100,000 [1] and a peak incidence in the fourth to sixth decades of life [2, 3]. Despite differences in origin and prognosis, their similar anatomical locations, clinical presentation and radiological findings often lead the two conditions to be considered together [4]. Chordomas arise from the remnants of the primitive notochord at the spheno-occipital synchondrosis, in comparison to chondrosarcomas which arise from mesenchymal cells or the embryonic rest of the cartilaginous matrix of the cranium [4]. Histological distinction can be made with immunohistochemistry as chordoma is positive for epithelial markers cytokeratin and epithelial membrane antigen (EMA), whilst chondrosarcomas are negative for both [5]. Radiologically, chordomas tend to be centred on the clivus [6], with chondrosarcomas typically centred on the petro-occipital fissure [5]. Diffusion-weighted MRI may aid diagnosis [7]. Both tumours tend to be locally invasive, and, given their proximity to the important structures of the skull base, they can present a serious management challenge.

Due to their rarity and the limitations of the published literature to case series, detailed risk stratification and prognostic data are not available. However, prognosis is reasonable with 5-year survival of 65% for chordomas and 81.8% for chondrosarcomas [8]. Surgery is the main treatment modality with a number of studies suggesting that maximising resection reduces risk of recurrence and improves survival [9]. Complete resection is challenging due to the locally aggressive nature of these tumours and the close proximity to important skull base structures. Adjuvant post-operative radiotherapy is usually administered, with a further reduction in recurrence [10, 11]. This may take the form of fractionated high-dose photon therapy [12], proton beam therapy (PBT) [13] or hypofractionated stereotactic [14] modalities.

We are able to present our 9-year consecutive case series of skull base chordomas and chondrosarcomas from a UK tertiary referral centre, discussing the treatments offered and outcomes, including survival, recurrence and radiotherapy complications. As far as we are aware, our case series includes the largest number of UK skull base chordomas and chondrosarcomas managed surgically via a transnasal endoscopic approach. This analysis was carried out to improve understanding around current treatment options and to better inform the management of future patients.

## Methods

Consecutive case series of all skull base chordomas and chondrosarcomas managed at a tertiary referral centre in the UK, over a 9-year period (2007–2016). Analysis was performed retrospectively by review of the electronic skull base multidisciplinary team (MDT) database and the digital patient record system at our centre. The above time period covers when the patients were discussed at MDT. Data was extracted by a single author (JM) and entered into a spreadsheet for analysis. Where gaps in the data were identified, further information was sought through contact with the clinician in charge of that patient’s care. This included an ENT surgeon, a neurosurgeon and a consultant oncologist. Data extracted is summarised in Table 1.

**Table 1:** Data collected

Patient age	
Diagnosis	
Details of surgery	
Adjuvant radiotherapy (PBT or TomoTherapy)	
PBT dose	
G3/4 toxicity (as per Common Terminology Criteria for Adverse Events (CTCAE, v4) [13]	
Local recurrence/pattern of relapse	
Survival	

## Results

A total of 24 patients were identified (11 chordomas, 13 chondrosarcomas) with a mean age of 52 years (range 21–91). All patients underwent surgical resection, and seven patients required more than one operation, excluding initial biopsy. During this period, all patients were considered for adjuvant radiation including using proton beam therapy (PBT) via the National Health Service programme accessing treatment abroad. All but two patients had adjuvant radiotherapy (19 had PBT, two had intensity modulate radiotherapy (IMRT) using TomoTherapy locally and one patient moved abroad for adjuvant therapy but no further treatment details were available). Results have been divided into chordomas and chondrosarcomas below.

### Chordomas

Eleven patients were identified with a mean age of 53 years (range 23–81). All chordoma patients underwent surgical resection via a transnasal endoscopic approach. Four patients required more than one operation, excluding initial biopsy. Nine patients went on to have adjuvant PBT, and one patient had IMRT. Five patients treated with proton therapy had confirmed radiological recurrence of their chordoma, all five of which were local. The first of these patients underwent stereotactic radiosurgery 3 years after her initial treatment and remains alive 5 years after this; however, she now has further disease progression. The second patient relapsed 4 years after initial treatment. This was treated with a craniotomy and further resection without adjuvant radiotherapy, and the patient is now alive and well 4 years later. The third patient underwent further surgical resection and received adjuvant afatinib; they remain alive but have further disease progression. The fourth patient underwent further surgery and remains stable, despite residual disease. The final patient relapsed 3 years after initial treatment and died within a month of diagnosis having had no further treatment. The patient treated with surgery and IMRT did not relapse but died of a cause unrelated to their chordoma 4 years post treatment. Mean duration of follow-up following last treatment was more than 7 years. One patient had moved abroad for adjuvant radiotherapy, and no further treatment details were available. Of the eight patients with at least 4 years follow-up there were two mortalities giving a 4-year survival rate of 75%. Chordoma patient treatments and outcomes are summarised in Table 2.

**Table 2:** Treatment and outcome summary for chordoma patients

Patient	Diagnosis	Initial treatment	Adjuvant therapy	Disease recurrence/progression	Further treatment	Survival	Follow-up from initial treatment
1	Clival chordoma	Transnasal endoscopic resection	PBT	Yes	Surgery + stereotactic radiosurgery	Yes	10 years
2	Clival chordoma	Transnasal endoscopic resection	PBT	Yes	Surgery + afatinib	Yes	8 years
3	Clival chordoma	Transnasal endoscopic resection	PBT	Yes	No	No	4 years
4	Clival chordoma	Transnasal endoscopic resection	PBT	Lost to follow-up due to CVA post PBT	-	-	3 years
5	Clival chordoma	Transnasal endoscopic resection	PBT	No	No	Yes	6 years
6	Clival chordoma	Transnasal endoscopic resection	PBT	Yes	Surgery	Yes	4 years
7	Clival Chordoma	Transnasal endoscopic resection	PBT	No	No	Yes	4 years
8	Clival chordoma	Transnasal endoscopic resection	PBT	Yes	Surgery	Yes	4 years
9	Clival chordoma	Transnasal endoscopic resection	PBT	No	No	Yes	3 years (lost to follow-up beyond this)
10	Clival chordoma	Transnasal endoscopic resection	Nil	-	-	-	Lost to follow-up post operatively
11	Clival chordoma	Transnasal endoscopic resection	IMRT	No	No	No unrelated to disease	4 years

### Chondrosarcomas

Thirteen patients were identified with a mean age of 50 years (range 21–91). All 13 patients underwent surgical resection, and seven patients required more than one operation, excluding initial biopsy. Ten patients went on to have adjuvant PBT, and two patients had IMRT. Only one patient had disease recurrence following PBT, which was at the margin of the PBT field. This patient had initially been treated with surgical debulking alone with proton therapy as adjuvant treatment to a second debulking 3 years after the first. He underwent a third debulking 3 years after this. The patient ultimately died 10 years after initial diagnosis and treatment of complications relating to disease and treatment. One of the two patients treated with surgery and TomoTherapy developed local recurrence 10 years later which was treated with endoscopic resection and remains alive and disease free 5 years after this. The patient that did not develop recurrence is alive and well 9 years post treatment. One patient had no adjuvant radiotherapy. They were an elderly patient that underwent radical surgery but was felt to be too frail to undergo radiotherapy, following MDT discussion. Despite residual disease, this patient is functioning well 6 years on from surgery. Of the 12 patients with at least 5 years follow-up, there was one mortality giving a 5-year survival rate of 91.6%. Chondrosarcoma patient treatments and outcomes are summarised in Table 3.

**Table 3:** Treatment and outcome summary for chondrosarcoma patients

Patient	Diagnosis	Initial treatment	Adjuvant therapy	Residual disease	Disease progression/recurrence	Further treatment	Survival	Follow-up from initial treatment
1	Left petrous apex chondrosarcoma	Open surgery	PBT	Yes	No	No	Yes	12 years
2	Left maxilla chondrosarcoma	Open surgery	No	No	Yes	Surgery + PBT	Yes	16 years
3	Petroclival chondrosarcoma	Open surgery	PBT	Yes	No	No	Yes	11 years
4	Clival chondrosarcoma	Transnasal endoscopic resection	PBT	Yes	No	No	Yes	10 years
5	Anterior skull base chondrosarcoma extending into left orbit	Transnasal endoscopic resection + open surgery	PBT	Yes	Yes	Further surgery	No	9 years
6	Lateral skull base chondrosarcoma	Open surgery	PBT	Yes	No	No	Yes	10 years
7	Cavernous sinus chondrosarcoma	Open surgery	PBT	Yes	No	No	Yes	8 years
8	Cavernous sinus chondrosarcoma	Transnasal endoscopic resection + open surgery	PBT	Yes	Yes	Further endoscopic resection	Yes	10 years
9	Dorsum of sella chondrosarcoma	Transnasal endoscopic resection	PBT	Yes	No	No	Yes	7 years
10	Central skull base chondrosarcoma	Surgery abroad—details unknown	PBT	No	No	No	Yes	23 years
11	Paracavernous/petrous apex chondrosarcoma	Open surgery	Nil	Yes	No	Further surgery	Yes	9 years
12	Petrous bone chondrosarcoma	Open surgery	IMRT	No	No	No	Yes	3 years
13	Petroclival chondrosarcoma	Open surgery	IMRT	Unknown	Yes	Transnasal endoscopic resection	Yes	15 years

### Complications

One patient’s surgical resection for chordoma was complicated by recurrent post-operative infections and subsequent haemorrhage leading to CVA, requiring a decompressive craniotomy and shunt insertion for hydrocephalus. A further patient required repair of a CSF leak at a second procedure 6 weeks after initial surgery when further residual chordoma was resected. Late radiation toxicity was carotid artery stenosis leading to significant permanent disability (1) and temporal lobe necrosis (1) following PBT for the chordoma cohort. For the chondrosarcoma cohort, late radiation toxicity was temporal lobe necrosis (2), one associated with epilepsy, and osteoradionecrosis (1) in patients receiving PBT. Three patients developed either new or worsening of pre-treatment cranial nerve palsies.

## Discussion

We present our 9-year consecutive case series of skull base chordomas and chondrosarcomas from a UK tertiary referral centre. As far as we are aware, our case series includes the largest number of UK skull base chordomas and chondrosarcomas managed surgically via a transnasal endoscopic approach. Choi et al. [3] have published a larger case series which includes 97 patients with chordomas of the craniocervical junction, between two centres in the UK and Germany (1982–2007); however, most of their patients underwent resection via a transoral or extended transoral approach, with no endoscopic cases. They do not comment on the use of adjuvant radiotherapy. In terms of surgical planning, they advocate radical resection early to improve outcomes. They also acknowledge the unique challenges associated with such tumours, both in terms of biology and anatomical location, and as such, we agree with their recommendation that these tumours should be managed at specialised centres via the skull base multidisciplinary team. They report a 5-year survival of 55%, compared to 77.8% in our series, albeit with a much larger cohort.

Chordomas have a tendency to arise from the middle and upper third of the clivus, an area that can be difficult to access surgically and one that lies in immediate proximity to critical neurovascular structures such as the carotid artery, brainstem and lower cranial nerves. All of our chordomas were managed using an expanded endoscopic endonasal approach. This technique is well established [15, 16] and utilises the nasal airway as a direct route to the skull base, with the endoscope providing narrow corridor access. The magnification improves visualization, allowing accurate dissection of the tumour and facilitating maximal resection, a principle central to the management of such tumours. This is enhanced by the use of ultra-high-definition monitors. The use of various rigid endoscopes, ranging from 0 to 70°, further improves exposure for the operating surgeon.

Surgical complication rates in this study were relatively low in the context of these disease processes. Our CSF leak rate of 4.2% (1 patient from 24) is comparable to the 6.2% CSF leak rate reported by Choi et al. [3]; however, this is unlikely to reach statistical significance given the small number of patients. Other complications were rare, and anecdote suggests morbidity is more likely to be associated with adjuvant radiotherapy.

It is well established that complete resection of skull base chordomas and chondrosarcomas is difficult, and, even in cases with good macroscopic clearance, there may be microscopic tumour cells left behind. This is demonstrated by the high recurrence rate in our series, particularly in the chordoma cohort, and this is consistent with current literature [17]. As a result, many patients can be offered higher dose adjuvant radiotherapy which provided the best chance of local control. Excellent local control rates with PBT are reported in the literature [18]. Historically, the main advantage of using PBT over conventional photon therapy around the skull base was the ability to deliver a higher dose of radiation close to critical radiosensitive organs. However, the precise advantage over modern photon techniques remains uncertain. Regardless, it is important that surgical planning takes into account the ability to deliver high-dose radiation post resection.

## Data Availability

Data held on the electronic skull base multidisciplinary team (MDT) database and the digital patient record system at our centre
